# 

**Published:** 1883-04-20

**Authors:** 


### TABLE OF CONTENTS.

ORIGINAL AND SELECTED ARTICLES.

Diphtheria—Treatment of, by Addison C. Fox, M. D., of Maryland, 121. Some Practical Facts Relating to the Trance State in Inebriety, by T. D. Crothers, M. D., 122. Hypodermic Use of Stimulants, by T. Curtis Smith, M. D., Aurora, Ind., 127. Varicocele, by A. J. Howe, M. D., 130. Case of the Puerperal Breast—Gaunt, 132. Revivification, by S. Waterman, M. D., of New York, 134. Dislocation of the Shoulder, 136. Treatment of Piles, by J. G. Westmoreland, M. D., Atlanta, Ga., 137.

ABSTRACTS AND GLEANINGS.

Quackery Years Ago, 138. Chronic Ovaritis, 139. Rape During Hypodermic Slumber, 141 Treatment of Eczema of the Genitalia, Pruritus and Leucorrhœa; Sponge-Grafting; Iodide of Potassium in Frontal Headache, 142. Dislocations of the Thigh Reduced, etc.; Medical Education of Women in Russia, 143. Perils of Medical Practice, 144. The Bacillus of Whooping-Cough, 145. Veratrum and Arsenic in the Treatment of Phthisis; Reliquet's Injection for Chronic Cystitis, 146. The Value of Cannabis Indica in Checking Epistaxis; Chamomile in Infantile Diarrhœa; Aconite and Belladonna in Erysipelas, 147. The Bacilli of Tubercle only Fat-Crystals; Gelseminum Sempervirens in Tetanus; A New Vegetable Styptic, 148. Whooping-Cough; Calomel in Diphtheria; Pop-corn a Remedy for Vomiting of Pregnancy, 149. The Cure of Saccharine Diabetes; A Bar to an Action for Malpractice; Chest Development by Exercise, 150.

SCIENTIFIC ITEMS.

Living a Double Life; The Hygiene of Shoes, 151. Science and Christianity; One by one; The Electric Light; A Submarine Vessel, 152.

PRACTICAL NOTES AND FORMULÆ.

Anti-Diphtheritic Inhalations; Gonorrhœa, 153. In Anorexia; Elixir of Salicylic Acid; Cosmetic Preparations, 154. A Cholagogue; Turpentine for Tænia Solium; New Compound Cathartic Pill, 155.

EDITORIALS AND MISCELLANEOUS.

Editorial Notices, 156. Mental Diseases, 157. Sensible; The Good of Medical Societies; Heavy, 158. Association of American Medical Editors; Books and Pamphlets Received; Receipted, 159. Special Notices 160.

